# Adverse Childhood Experiences and Building Resilience With the JoyPop App: Evaluation Study

**DOI:** 10.2196/25087

**Published:** 2021-01-04

**Authors:** Angela MacIsaac, Aislin R Mushquash, Shakira Mohammed, Elizabeth Grassia, Savanah Smith, Christine Wekerle

**Affiliations:** 1 Lakehead University Thunder Bay, ON Canada; 2 McMaster University Hamilton, ON Canada

**Keywords:** adverse childhood experiences, resilience, emotion regulation, smartphone, app, childhood, emotion, mental health, transition, innovation, intervention

## Abstract

**Background:**

The effects of adverse childhood experiences (ACEs) on mental health, self-regulatory capacities, and overall resilience are well-known. Given such effects, ACEs may play a role in how individuals adjust to challenges later in life. Of interest in this study is the transition to university, a time of heightened stress when adapting to circumstances is required and when those with ACEs may need additional in-the-moment support to exercise resilience. A smartphone app may provide a worthwhile and readily accessible medium for a resilience intervention, provided behavioral outcomes are adequately evaluated.

**Objective:**

This study evaluates the impact of an innovative, smartphone app–based resilience intervention. The JoyPop app was designed to promote resilience through the use of self-regulatory skills such as emotion regulation and executive functioning. Among a sample of first-year undergraduate students, we explored whether use of the app would be associated with positive changes in resilience and related outcomes, and whether these benefits were influenced by level of childhood adversity.

**Methods:**

Participants (N=156) were requested to use the JoyPop app for 4 weeks, at least twice daily. Changes in resilience, emotion regulation, executive functioning, and depression were assessed after 2 and 4 weeks of app usage using multilevel modeling.

**Results:**

The sample of 156 participants included 123 females and 33 males, with a mean age of 19.02 years (SD 2.90). On average participants used the app on 20.43 of the possible 28 days (SD 7.14). App usage was associated with improvements in emotion regulation (*χ*^2^_1_=44.46; *P*<.001), such that it improved by 0.25 points on the 18-point scale for each additional day of app usage, and symptoms of depression (*χ*^2^_1_=25.12; *P*<.001), such that depression symptoms were reduced by .08 points on the 9-point scale with each additional day of app usage. An interaction between ACEs and days of app usage existed for emotion regulation, such that participants with more adversity evidenced a faster rate of change in emotion regulation (*P*=.02).

**Conclusions:**

Results highlight that daily incorporation of an app-based resilience intervention can help youth who have experienced adversity to improve emotion regulation skills and experience reductions in depression. The JoyPop app represents an important step forward in the integration of resilience intervention research with a technology-based medium that provides in-the-moment support.

## Introduction

### Adverse Childhood Experiences

Adverse childhood experiences (ACEs) have profound and long-lasting effects on a broad spectrum of physical and psychological health outcomes. While the assessment of ACEs has changed somewhat since the seminal study by Felitti et al [1], it is well-recognized that the 10 established categories of childhood adversity (divided into household dysfunction and child maltreatment exposures) are associated with mental health difficulties that can last well into adulthood [2-6]. A dose-response relationship exists, such that risk accumulates with each one-unit increase in ACEs [2]; further, categorically, those with high ACEs (eg, 4 or more in adults) are at a significantly greater risk for mental health issues compared to those with low or no ACEs [3,6]. These impacts have remained consistent across cohorts dating back to the 1900s [7]; thus, the impacts of ACEs are seemingly immune to societal, cultural, or health-related changes but instead represent robust effects on mental health and well-being.

### The Mechanism of Action of ACEs

While a moderate amount of stress can support the development of coping skills, ACEs may constitute stress that is too overwhelming for an individual’s current regulatory processes [8]. Further, some ACEs involve removal of opportunities to learn these regulatory processes, such as in the case of neglect when appropriate self-regulation behaviors are not being modeled by the caregiver. Together, these experiences may lead to disrupted development of corresponding stress-sensitive brain areas [9-12], the results of which are observed behaviorally. For instance, those with ACEs may experience difficulties with emotion regulation, which are associated with a range of negative outcomes such as difficulties with alcohol and interpersonal relationships [13,14]. In addition to emotion regulation, impacts on self-regulation in the form of executive functioning are observed. Those with ACEs evidence deficits in a wide range of executive functions [15] that also impact well-being, resulting in difficulties such as mental health problems [16].

### The Transition to University

Life transitions often represent a period of increased challenges and associated stress, since one’s environment is in a state of flux and demands that individuals successfully adapt. The transition to university is worth examining as it is common among youth. In Canada, 916,944 youth aged 18-24 enrolled in university for the 2017-2018 school year [17]. In the United States, over 2,000,000 individuals made the transition to university or college in 2016 [18]. While the transition to university itself does not constitute adversity and is instead a time of new and exciting opportunities, it is also marked by stress and an increased prevalence of mental health difficulties. A systematic review spanning two decades of research found the average prevalence of depression in students was 30.6%, much higher than in the general population [19]. Well-being and anxiety may also worsen during university [20]. Surely, the stress of university is not specific to only those with ACEs, rendering it a period where the successful coping of any student is of the utmost importance. Where those with a history of childhood adversity are at a disadvantage, however, is in lacking the resources to cope; those with ACEs may need extra support in exercising resilience during the transition to university.

### Resilience

Those with ACEs may be doubly disadvantaged compared to peers when coping with the increased stress associated with the transition to university. Specifically, ACEs serve to challenge one’s capacity for resilience. Resilience refers to the capability, resources, and processes available to a person or system to adapt successfully in the face of adversity [21,22]. The operationalization of resilience has changed over time, progressing from focusing on individuals who seemingly possess a unique quality of invulnerability, to variables explaining resilient individuals, to a more recent developmental systems view, whereby resilience is seen as the result of dynamic interactions of various systems (eg, biological and sociocultural systems) [21,22]. An implication of this shift is that resilience is seen as a result of ordinary survival processes common to humans as adaptive creatures, including self-regulatory skills like emotion regulation and executive functioning [21-23]. Moderate amounts of stress allow one to learn such self-regulatory skills and exercise resilience in the future, whereas stressors that are too challenging, such as some ACEs, overwhelm the individual and increase the risk of negative outcomes in the future [24].

Such self-regulatory skills like emotion regulation and executive functioning are vital underlying processes of resilience. In a systematic review of adolescents and young adults, Fritz et al [25] found that components of emotion regulation and executive functions were included among 13 of 25 resilience factors supported by research. Many studies have implicated emotion regulation and executive functioning in the relationship between adversity and overall resilience, studying these abilities as moderators or mediators of resilience outcomes [16,26-28], or examining the separate influence of both self-reported resilience (eg, the Connor-Davidson Resilience Scale [29]) and self-regulation on later mental health outcomes [14]. In recognition of the myriad of ways resilience is operationalized methodologically, we see examining it across various domains, including underlying processes and self-reported resilience, as important. If resilience results when normal adaptive capacities like self-regulation are properly promoted [21], resilience may be restored by the same mechanism [22]. Experts have highlighted a need for resilience supports external to the individual [30,31]. Moreover, it is futile to screen for ACEs in clinical practice without being able to respond with appropriate interventions [32]. The transition to university may be an opportune time to promote resilience because although it is characterized by increased stress, it may be appropriately challenging such that one can learn to exercise resilience if assisted [24]. Moreover, the movement from adolescence into young adulthood is a time when many of those who struggled early on can move onto more positive paths [21] and when self-regulatory brain regions are still developing [33]. Practically, students would benefit from having strategies available for managing emotion that then become part of their self-regulatory skill repertoire in exercising resilience against the stress of the transition.

Advances in technology have opened doors for implementing this kind of resilience intervention [21]. This includes the use of smartphone apps. The potential of a smartphone app to act as a resilience-promoting tool lies in the readily accessible nature of smartphones, meaning support can be accessed in the moment when it is needed [34]. There is a growing body of empirical literature supporting digital health interventions, including mobile phone apps, as facilitators of improved health and well-being. Specifically, recent evidence supports the use of various digital health interventions targeting coping, stress reduction, self-management skills, and symptom reporting in improving medication and treatment adherence, health knowledge, and anxiety in those with chronic health conditions [35-37]. Other app-based interventions have demonstrated success in improving mental health–related outcomes such as depression, stress, and substance use [38]. Mental health–focused apps may be a cost-effective way of providing psychological support to a wider population who may not otherwise have access to formal interventions [39]. Among university students, smartphone use is ubiquitous, and students have demonstrated a willingness to engage with smartphone health-related apps [40]. At the same time, researchers have described the gap between the incredible number of apps and the demonstration of their safety and efficacy [34]. For example, Donker et al [38] found only 8 studies that assessed outcomes of apps using a pre-post design or control group of the 5464 abstracts they searched. Similarly, in their review, Lui et al [41] concluded there is not enough evidence for the effectiveness of any one individual mental health app and that it was unknown whether there were any adverse effects associated with existing apps. Instead of behavioral outcomes, studies more often focus on aspects of accessibility and usability [42]. With respect to university students specifically, most app studies have examined smoking and alcohol cessation, thus presenting a need for a broader focus across other issues experienced by this population [40].

### Aims of This Study

Although the detrimental effects of ACEs on the self-regulatory functions that underlie resilience are well-known, a gap exists in translating these findings into evidence-based interventions that promote resilience in youth with ACEs. The aim of this study was to test whether a smartphone app (JoyPop) [43] promotes resilience over time among youth with varying degrees of ACEs who are navigating the transition to university. Practically, we theorized that the JoyPop app would benefit those transitioning to university by helping them identify, reflect on, and regulate their emotions and improve executive function skills more broadly, contributing to overall resilience against the stress of the university transition. Further, the ease of access of a smartphone app allows the practical benefit of receiving support as needed, which was thought to be conducive to regular usage and the skills becoming routine over time. Resilience was examined across multiple domains, including self-reported resilience and improvement of self-regulatory functions. We also examined a negative outcome of relevance to university students (depression) to ensure the app did not have any unexpected adverse effects, consistent with the gaps identified by Lui et al [41]. More specifically, we examined whether there was a significant rate of change over app usage in these resilience-related outcomes, as well as the relationship between the rate of change and ACEs score. We hypothesized that using the JoyPop app would be associated with a positive rate of change in resilience-related outcomes, with a direct relationship between the number of days the app was used and the amount of change observed, and that this would be most evident among those with higher ACEs scores as they have more to gain from a resilience-promoting intervention focused on bolstering self-regulatory skills.

## Methods

### Participants and Procedure

This study was approved by the Research Ethics Board at Lakehead University. Students were eligible if they were first-year undergraduate students, owned an iPhone, and were fluent in English. Data were collected in waves over the duration of the school year (both fall and winter semesters). Participants attended 3 laboratory sessions which were run in a group format (pre-app, mid-app [after 2 weeks of app usage], post-app [after 4 weeks of app usage]). Pre-app sessions were run by authors AM, SM, and EG, while mid-app and post-app sessions were run by these authors or supervised research assistants. During the pre-app session, participants received information about the study, provided informed consent, and then were guided through downloading the JoyPop app and provided with a demonstration of all the features. Participants were asked to use the JoyPop app at least twice per day over 4 weeks; no additional requirements were made with respect to feature usage or time spent using the app. Participants returned to the laboratory for mid-app and post-app group sessions, during which time they were requested to complete the self-report measures. Each morning and evening (ie, twice per day), participants were sent reminder emails to use the app. They also received reminder emails to attend their laboratory sessions. If participants encountered any technical difficulties while using the app, they were encouraged to contact the research team, who would liaise with the app development company to resolve any issues. Participants were provided with contact information for mental health supports in the event that they felt distressed at any point throughout their participation in the study. For completing the study, participants received CAD $90 (US $70) in cash or CAD $60 (US $47) and two bonus points toward an eligible psychology course.

### JoyPop App

**Figure 1:**
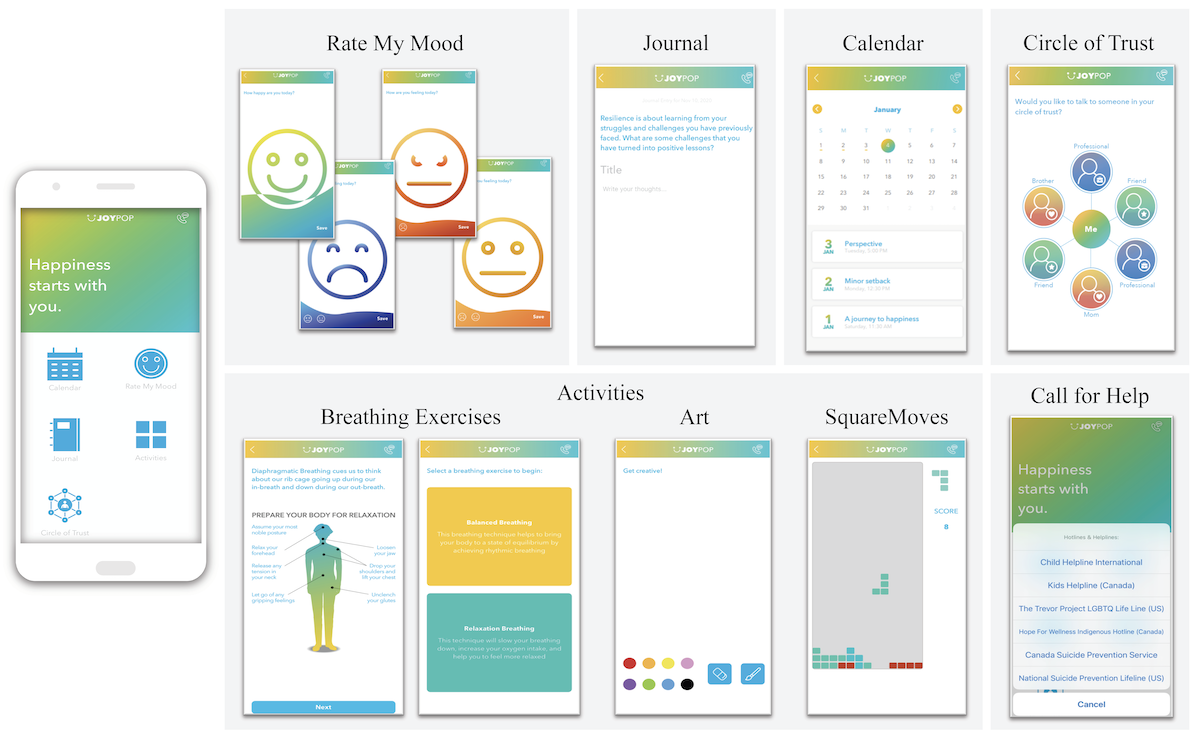
JoyPop app features.

The JoyPop app (see Figure 1 and Table 1) was developed from a cumulative research and parallel consultation approach. Findings from epidemiological and clinical research projects conducted by a federally funded Canadian team highlighted the importance of addressing the role of self-regulation in the link between adversity and mental health outcomes [44] and the resilience value of increasing self-reflection and self-regulation by fostering well-being [45]. This research also suggested there was value in developing internal assets (eg, positive identity [46], well-being [45], self-compassion [47,48]) to support self-reflection and self-regulation. Consultation with youth, service providers, and clinician-scientists informed design and discovery exercises with app development company Clearbridge Mobile. App features were consequently developed to target daily self-regulation via evidence-based techniques. For example, the Rate My Mood feature was designed to encourage attention towards positive (versus negative) mood states and help users understand and manage their emotions [49]. The Breathing Exercises support self-regulation and decreased physiological arousal [50]. The Journal feature was included in light of the long-term positive benefits evidenced by expressive writing [51], especially when writing is positively focused to foster self-regulation [52]. SquareMoves (a Tetris-like game) was included as activities of this nature can induce a “flow” state, a form of self-regulation that relinquishes negative self-focus [53]. The Art feature provides an opportunity for unrestricted creativity and is supported by positive benefits in memory and emotional expression through doodling [54]. The JoyPop app also includes connections with one’s support network (eg, the Circle of Trust feature) or established helplines (eg, through the telephone icon on the launch screen). Following the initial development of the app and associated features, youth involved with child-welfare and victim services as well as providers working closely with youth reviewed and provided direction on the final app features and functionality. Additional information on the development of the JoyPop app and the various features is available online [55] and in Figure 1 and Table 1.

**Table 1:** JoyPop app features and associated functions.

JoyPop feature	Function
Rate My Mood	Initially prompts users to rate their happiness by sliding a wave of colour up or down to indicate their happiness level. If happiness is rated at 50% or above, the user receives a thumbs up icon and a motivational quote. If happiness rating is lower than 50%, the user is prompted to rate how sad, angry, or “meh” they are feeling using the same technique. Once users have rated their negative emotion, they are given a motivational quote and a prompt to complete an activity from the app.
Journal	Allows the user to complete a journal entry by entering their free-flowing thoughts and emojis or by responding to a resilience-oriented writing prompt at the top of the screen. Users can save their journal entries to the Calendar feature.
Calendar	Allows the user to reflect on previously saved journal entries by date.
Circle of Trust	Allows the user to input up to 6 safe social contacts (ie, by entering their name and phone number) to call if they want to talk or are in need of support. The user can label the contact as a friend, family member, or professional.
Breathing Exercises	Opens to a diagram of the body, with best-practice tips to prepare for relaxation. The user is then prompted to choose between completing a balanced breathing exercise (rhythmic breathing) or a relaxation breathing exercise (slowed breathing). Once the user selects which breathing exercise they would like to complete, they are guided through the breathing exercise with text instructions and an animated diagram.
Art	Allows the user to doodle in colour, swiping their finger across the screen as the paint brush.
SquareMoves	A game in which multi-shaped blocks fall from the top of the screen and the user taps on the shapes to rotate them or swipes them across the screen to move them as they fall to the bottom. Similar to the popular game Tetris, the objective is to form a solid line at the bottom of the screen (with no gaps). Once a line or multiple lines are formed, the blocks in the line break apart and the user is awarded points.
Call for Help	Allows the user to select a 24-hour helpline to call if they are experiencing distress while using the app. The user is provided with culturally specific Canadian and American hotlines (eg, an Indigenous-specific crisis line, LGBTQ helpline) to choose from.

### Measures

#### Adverse Childhood Experiences (ACEs) Questionnaire

A 10-item ACEs Questionnaire [1,56] was used to assess the occurrence of abuse, neglect, and household dysfunction during childhood. This questionnaire was only administered during the pre-app session as responses were not expected to change across time. Items were assessed at the category level as opposed to the level of individual event, and response options were “Never,” “At least once,” and “Many times” [56]. For this study, an answer of “At least once” or “Many times” was coded as 1, indicating the presence of the category. Previous ACEs questionnaires have assessed domestic violence only against one’s mother or stepmother and not that against one’s father or stepfather, potentially excluding adverse experiences of a similar nature. We assessed for domestic violence against either of these parental figures in the item focused on domestic violence. The ACEs Questionnaire demonstrates good test-retest reliability [57] and correlates with an inventory of lifetime traumatic exposure, demonstrating construct validity (*r*=0.69) [56]. In our sample, this measure had a Cronbach α coefficient of .78, indicating acceptable internal consistency.

#### Connor-Davidson Resilience Scale–10

The Connor-Davidson Resilience Scale–10 (CD-RISC-10) is a 10-item version of the original 25-item CD-RISC [29] designed to measure resilience as grounded in biological, psychological, and social facets [58]. Scores on this scale have demonstrated change in response to intervention [29,59], rendering this measure fitting for our purposes. The revised version correlates highly at *r*=0.92 with the original measure [29,60]. Items are rated on a 5-point Likert-type scale from “0 – Not true at all” to “4 – True nearly all the time”. In undergraduate students, the CD-RISC-10 demonstrated good internal consistency with a Cronbach α of .85, as well as good construct validity [29]. Cronbach α values in our study were .78, .86, and .90 during the pre-app, mid-app, and post-app sessions.

#### Executive Functioning Index

The Executive Functioning Index is a self-report measure of executive functioning that was created to sample a wide domain of executive functions and to assess the general adult population as opposed to clinical populations [61]. It is a 27-item measure with items rated on a 5-point Likert-type scale from “1 – Not at all much” to “5 – Very.” In this study the total score was used. This measure correlates with other measures of executive functioning [61] and predicts impulsive behaviors in delay discounting tasks [62]. Acceptable reliability was demonstrated with a Cronbach α of .82 in the original study [61] and .73, .77, and .76 during the pre-app, mid-app, and post-app sessions of this study.

#### Difficulties in Emotion Regulation Scale – Short Form

The Difficulties in Emotion Regulation Scale – Short Form (DERS-SF) [63] is a short-form version of the original DERS [64], which measures emotion regulation deficits. This short-form version retains the factor structure of the original measure and correlates highly with it [63]. The DERS-SF consists of 18 items rated on a 5-point Likert-type scale ranging from “Almost Never (0%-10%)” to “Almost Always (91%-100%).” Both the original and short-form scales contain 6 subscales; however, a total score can also be calculated and evidences good reliability and validity [63]. The total score was used in this study, and Cronbach α coefficients demonstrated good reliability at .89 across all timepoints.

#### Patient Health Questionnaire–9

The Patient Health Questionnaire–9 (PHQ-9) [65] is a self-report measure assessing the diagnostic criteria for depression. It is also used as a general severity measure, as total scores range from 0 to 27 [65]. Construct validity is demonstrated through associations with quality of life, health care utilization, and symptom-related difficulties [65]. In a study of medical patients, Cronbach α values were excellent at .86 and .89 [65], and our sample paralleled these findings with values of .88, .87, and .89 at pre-app, mid-app, and post-app sessions, respectively.

### Analytic Plan

Data analyses were conducted using Stata (IBM Corporation). Missing items within questionnaires were imputed with person-mean imputation, while missing data resulting from missing a session were accounted for using maximum likelihood estimation. While person-mean imputation tends to inflate reliability estimates, the risk of this is tolerable if the number of people with missing data and the missing data within each person’s measure are less than 15%-20% [66,67]. Maximum likelihood estimation is a preferred method of handling missing data in longitudinal designs and results in relatively unbiased parameters and valid model fit, performing similarly to multiple imputation [68,69]. An attrition analysis was conducted to compare those who attended all 3 sessions to those who missed at least 1 session on their age, sex, ethnicity, ACEs score, and all other pre-app outcome measures. Depending on the variable type, either a *t* test or chi-square test was used.

Multilevel modeling (MLM) was used to assess whether the JoyPop app confers improvements over time in resilience-related outcomes. MLM efficiently assesses longitudinal change while accounting for repeated measurements within the same person by structuring the data in a nested fashion [70]. Specifically, in longitudinal studies, individuals serve as the level 2 variable while time serves as the level 1 variable nested within individuals. Masten and Barnes [21] note that growth modeling (including MLM) represents a statistical advancement to answering questions regarding ACEs and resilience. While early studies separated analyses of person-centred and variable-centred focuses [71], the nested nature of MLM affords the ability to examine resilience-related variables while allowing the individual participant to be the level of analysis. A further departure from previous studies is that the relationship between ACEs score and resilience has been measured in a static fashion, whereas we sought to measure *change* in resilience.

Model building followed steps resembling those outlined by Peugh [72] for each of the four outcome variables separately. Multimedia Appendix 1 contains a description of this process, associated equations, and choice of parameter estimator and covariance structures. In total, two successive models were used for hypothesis testing. The first consisted of time as the only predictor. In this study, time was operationalized as the number of days the individual spent using the app, equal to 0 at pre-app for all participants and to a maximum of 14 and 28 assessed at the mid-app and study completion points, respectively. This approach allows time to vary for each participant, with some participants using the app less than daily or for the full 28 days, whereas using timepoint (pre-app, mid-app, post-app) as the time metric would mask individual differences in app usage [73]. The second model added ACEs score as a predictor to form the ACEs score × days of app usage interaction. A likelihood ratio test assessed whether the second model improved fit compared to the first. This was done for each of the four outcomes.

The interaction effects were interpreted in terms of the slope of days of app usage, that is, testing whether the slope of days of app usage was significantly different from 0 for each ACEs score. Benjamini-Hochberg corrections were used to correct for multiple comparisons; the false discovery rate for these corrections was set to 0.10 [73]. A power analysis was conducted using Monte Carlo simulations. Using parameters similar to the ones obtained in the analyses and a sample of 100, 500 repetitions of the simulation were run to assess power to detect a significant difference between a full model with interactions and a timepoint-only model. Resultant power was 0.92, indicating our final sample size of 156 participants was acceptable after accounting for attrition.

## Results

### Descriptive Statistics

**Figure 2:**
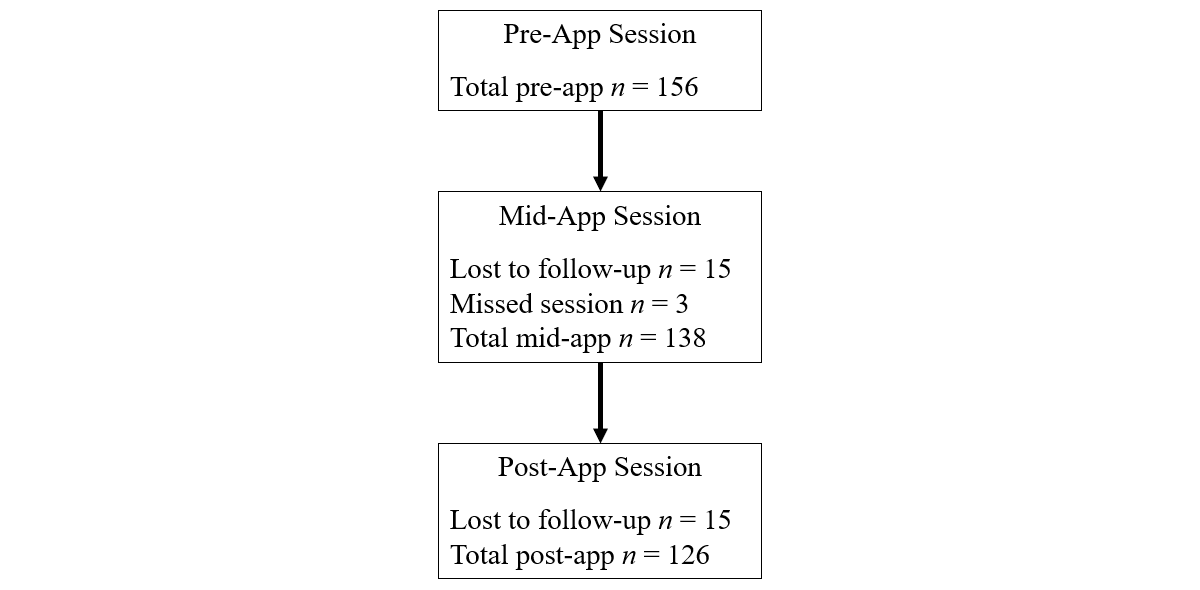
Flow of participant retention throughout the study.

The sample of 156 participants included 123 females (78.8%) and 33 males (21.2%). The mean age of the sample was 19.02 years (SD 2.90), with a range of 16 to 38. As expected, most of the sample consisted of adolescents (19 years or younger; 137, 87.8%) or youth (24 years or younger; 147, 94.2%) [74]. Of the participants, 109 (69.9%) identified as White, 18 (11.5%) as South Asian, 12 (7.7%) as Black, and the remaining 17 (10.9%) as East or Southeast Asian, Arab, Indigenous, or Latinx. Family income was assessed in categories that ranged from CAD $0-$19,999 (US $15,564) to greater than CAD $200,000 (US $155,649), with a median in the range of CAD $80,000-$99,000 (US $62,260-$77,046). Means and standard deviations for all measures at each timepoint are presented in Table 2. Of the 152 participants who reported on ACEs, 31 (20.4%) reported no ACEs, 25 (16.4%) reported one, 33 (21.7%) reported two, 19 (12.5%) reported three, 44 (28.9%) reported four or more; 4 participants did not provide a response. On average, participants used the app 20.43 of the possible 28 days (SD 7.14). Retention throughout the study was good: of the 156 who enrolled in the study initially, 138 completed the mid-app sessions (88.5%) and 126 (80.8%) completed the post-app sessions. These rates include 3 individuals who missed the mid-app session but then returned for the post-app session. Overall, 123 participants (78.8%) completed all 3 sessions. Figure 2 depicts retention of participants throughout the study. There were no statistically significant differences in age, sex, ethnicity, ACEs score, or other pre-app outcome measures between those who completed all 3 sessions and those who dropped out or missed the mid-app session. Excluding the missing data attributed to missed sessions, the percentages of missing questionnaire items across all participants and items at each timepoint (for which person-mean imputation was used) were as follows: 0.24% for the pre-app sessions, 0.16% for the mid-app sessions, and 0.27% for the post-app sessions. The majority of these missing data resulted from participants who missed one item in a multi-item questionnaire. As mentioned in our Analytic Plan above, the missing data resulting from a missed session were handled with maximum likelihood estimation.

**Table 2:** Descriptive statistics for study measures across each timepoint.

Measure		Mean (SD)
**ACEs^a^**		
	Pre-app	2.55 (2.17)
**CD-RISC-10^b^**		
	Pre-app	26.62 (5.95)
	Mid-app	27.49 (5.72)
	Post-app	27.63 (6.46)
**EFI^c^**		
	Pre-app	95.08 (11.43)
	Mid-app	94.50 (12.24)
	Post-app	95.63 (12.54)
**DERS-SF^d^**		
	Pre-app	42.91 (13.04)
	Mid-app	40.04 (12.59)
	Post-app	36.72 (11.40)
**PHQ-9^e^**		
	Pre-app	9.48 (6.09)
	Mid-app	8.80 (5.89)
	Post-app	7.52 (5.69)

^a^ACEs: Adverse Childhood Experiences Questionnaire.

^b^CD-RISC-10: Connor-Davidson Resilience Scale–10.

^c^EFI: Executive Functioning Index.

^d^DERS-SF: Difficulties in Emotion Regulation Scale – Short Form.

^e^PHQ-9: Patient Health Questionnaire–9.

### Multilevel Modeling

Table 3 contains the β values, standard errors, and confidence intervals for resilience. The model with days of app usage as the sole predictor was not significant. Adding ACEs score and the 2-way interaction between ACEs score and days of app usage did not further improve the model, and no predictors were significant, meaning that neither app usage nor one’s experience of childhood adversity was related to change in resilience.

Table 4 contains the β values, standard errors, and confidence intervals for executive functioning. The model with days of app usage as the sole predictor was not significant. Adding ACEs score and the 2-way interaction resulted in a significant effect of ACEs score at *P*=.002, such that higher ACEs scores were associated with lower executive functioning (*χ*^2^_3_=10.10; *P*=.02), but the 2-way interaction was not significant, meaning that app usage was not related to a change in executive functioning, nor was there a relationship between app usage and change in executive functioning that depended on one’s childhood adversity. This second model demonstrated improved fit to the model with days of app usage as the sole predictor according to a likelihood ratio test (*χ*^2^_2_=9.13; *P*=.01).

**Table 3:** Coefficients/estimates, standard errors (in brackets), and confidence intervals for the fixed effects of interest and random effects for the Connor-Davidson Resilience Scale–10 models.^a^

Parameter		DAU^b^ only, estimate (SE), 95% CI	*P* value	ACEs^c^ and DAU, estimate (SE), 95% CI	*P* value
**Fixed effects**					
	Intercept	26.81 (0.45), 25.92 to 27.70	<.001	27.66 (0.69), 26.30 to 29.02	<.001
	DAU	0.04 (0.02), 0.00 to 0.08	.08	0.05 (0.03), −0.02 to 0.11	.15
	ACEs	N/A^d^	N/A	−0.34 (0.21), −0.74 to 0.07	.105
	ACEs × DAU	N/A	N/A	0.00 (0.01), −0.02 to 0.01	.68
**Variance components**					
	Residual	11.95 (1.29), 9.68 to 14.76	N/A	11.94 (1.28), 9.68 to 14.73	N/A
	Intercept	21.81 (3.20), 16.36 to 29.09	N/A	21.25 (3.14), 15.92 to 28.38	N/A
	Slope	0.01 (0.01), 0.01 to 0.04)	N/A	0.01 (0.01), 0.01 to 0.04	N/A

^a^The number of observations in these models at level 1 (timepoint) is 418; at level 2 (participant) it is 155.

^b^DAU: days of app usage.

^c^ACEs: adverse childhood experiences.

^d^N/A: not applicable.

**Table 4:** Coefficients/estimates, standard errors (in brackets), and confidence intervals for the fixed effects of interest and random effects for the Executive Functioning Index models.^a^

Parameter		DAU^b^ only, estimate (SE), 95% CI	*P* value	ACEs^c^ and DAU, estimate (SE), 95% CI	*P* value
**Fixed effects**					
	Intercept	94.77 (0.91), 92.98 to 96.56	<.001	97.92 (1.37), 95.23 to 100.61	<.001
	DAU	0.03 (0.04), −0.02 to 0.10	.43	0.00 (0.06), −0.10 to 0.11	.95
	ACEs	N/A^d^	N/A	−1.24 (0.41), −2.04 to −0.43	.003
	ACEs × DAU	N/A	N/A	0.01 (0.02), −0.02 to 0.04	.55
**Variance components**					
	Residual	30.88 (3.56), 24.63 to 38.72	N/A	30.72 (3.53), 24.52 to 38.48	N/A
	Intercept	102.97 (13.55), 79.56 to 133.26	N/A	96.56 (12.83), 74.43 to 125.28	N/A
	Slope	0.04 (0.02), 0.02 to 0.12	N/A	0.05 (0.02), 0.02 to 0.12	N/A

^a^The number of observations in these models at level 1 (timepoint) is 416; at level 2 (participant) it is 155.

^b^DAU: days of app usage.

^c^ACEs: adverse childhood experiences.

^d^N/A: not applicable.

**Figure 3:**
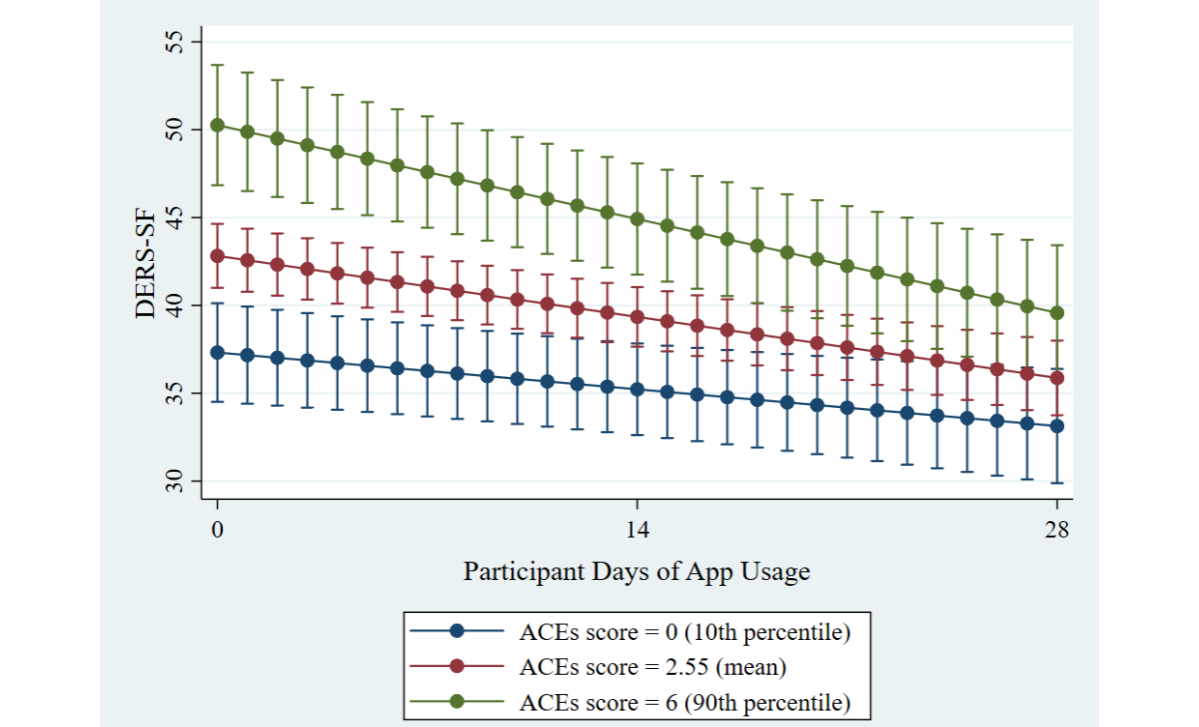
Changes in difficulties with emotion regulation across time and ACEs score. ACEs: adverse childhood experiences. DERS-SF: Difficulties in Emotion Regulation – Short Form.

Table 5 contains the β values, standard errors, and confidence intervals for difficulties with emotion regulation. Days of app usage was significant in the initial model (*P*<.001), with a Wald test for the model of *χ*^2^_1_=44.46, *P*<.001, such that difficulties with emotion regulation decreased by an average of 0.25 units on the 18-point scale with each additional day of app usage. Adding ACEs score and the 2-way interaction to the model, days of app usage, ACE score, and the 2-way interaction were significant at *P*=.009, *P*<.001, and *P*=.02, respectively, with a Wald test for the overall model of *χ*^2^_3_=71.22, *P*<.001. The 2-way interaction was such that difficulties with emotion regulation decreased at a higher rate the higher one’s ACEs score: when an individual had no ACEs, their difficulties with emotion regulation score decreased by 0.14 units with each additional day of app usage, but when an individual had an ACEs score of 6 (placing them in the 90th percentile), their score decreased by 0.38 units with each additional day of app usage (see Figure 3; slopes of lines are presented in Table 6). More specifically, for those with no reported childhood adversity, the marginal mean score at pre-app was 37.32, and this decreased to 33.13 when the app was used for 28 days, yet the pre-app marginal mean for those with a high ACEs score of 6 was 50.26 and decreased to 39.57 by 28 days of app usage. Table 6 contains the slopes of days of app usage for each ACEs score. A likelihood ratio test comparing this model to the model that only included days of app usage indicated increased fit (*χ*^2^_2_=24.22; *P<*.001).

**Table 5:** Coefficients/estimates, standard errors (in brackets), and confidence intervals for the fixed effects of interest and random effects for the Difficulties in Emotion Regulation Scale models.^a^

Parameter			DAU^b^ only, estimate (SE), 95% CI		*P* value		ACEs^c^ and DAU, estimate (SE), 95% CI		*P* value
**Fixed effects**									
	Intercept	42.81 (0.98), 40.89 to 44.74		<.001		37.32 (1.43), 34.51 to 40.13		<.001	
	DAU	−0.25 (0.04), −0.32 to −0.18		<.001		−0.15 (0.06), −0.26 to −0.04		.009	
	ACEs	N/A^d^		N/A		2.16 (0.43), 1.32 to 3.0		<.001	
	ACEs × DAU	N/A		N/A		−0.04 (0.02), −0.07 to −0.01		.02	
**Variance components**									
	Residual	48.72 (4.28), 41.01 to 57.87		N/A		47.86 (4.20), 40.30 to 56.84		N/A	
	Intercept	108.07 (14.78), 82.66 to 141.29		N/A		93.25 (13.01), 70.95 to 122.57		N/A	

^a^The number of observations in these models at level 1 (timepoint) is 417; at level 2 (participant) it is 155.

^b^DAU: days of app usage.

^c^ACEs: adverse childhood experiences.

**Table 6:** DERS-SF – Slopes, standard errors (in brackets), and confidence intervals of participant days of app usage for each ACEs score.

ACEs^a^ score	Slope of participant days of app usage, slope (SE), 95% CI	*P* value^b^
0	−0.15 (0.06), −0.26 to −0.04	.009
1	−0.19 (0.05), −0.28 to −0.10	<.001
2	−0.23 (0.04), −0.30 to −0.15	<.001
3	−0.27 (0.04), −0.34 to −0.19	<.001
4	−0.30 (0.04), −0.39 to −0.21	<.001
5	−0.34 (0.05), −0.45 to −0.24	<.001
6	−0.38 (0.07), −0.51 to −0.25	<.001
7	−0.42 (0.08), −0.58 to −0.26	<.001
8	−0.46 (0.09), −0.64 to −0.27	<.001
9	−0.50 (0.11), −0.71 to −0.28	<.001

^a^ACEs: adverse childhood experiences.

^b^After correcting for multiple comparisons using the Benjamini-Hochberg procedure, all significant *P* values remained significant at the .05 level.

Table 7 contains the β values, standard errors, and confidence intervals for depression symptoms. Days of app usage was significant as the sole predictor (*P*<.001), with a Wald test for the model of *χ*^2^_1_=25.12, *P*<.001, such that depression symptoms decreased by an average of 0.08 units on the 9-point scale with each additional day of app usage. Adding ACEs score and the 2-way interaction to the model, ACEs score was significant (*P*<.001), and days of app usage remained significant at *P*=.01, meaning that there was a positive conditional effect of ACEs score on depression symptoms and a negative conditional effect of days of app usage. However, the 2-way interaction between ACEs score and days of app usage was not significant, which indicates that the reduction in depression symptoms with increased days of app usage seen in the first model did not depend on one’s ACEs score. The model overall was significant at *χ*^2^_3_=51.72, *P*<.001, and a likelihood ratio test evidenced improved fit over the days of usage–only model (*χ*^2^_2_=24.31; *P*<.001).

**Table 7:** Coefficients/estimates, standard errors (in brackets), and confidence intervals for the fixed effects of interest and random effects for the Patient Health Questionnaire–9 models.^a^

Parameter		DAU^b^ only, coefficient (SE), 95% CI	*P* value	ACEs^c^ and DAU, coefficient (SE), 95% CI	*P* value
**Fixed effects**					
	Intercept	9.57 (0.47), 8.65 to 10.49	<.001	6.98 (0.68), 5.65 to 8.31	<.001
	DAU	−0.08 (0.02), −0.11 to −0.05	<.001	−0.06 (0.02), −0.11 to −0.01	.01
	ACE	N/A^d^	N/A	1.02 (0.20), 0.62 to 1.41	<.001
	ACEs × DAU	N/A	N/A	−0.01 (0.01), −0.02 to 0.01	.32
**Variance components**					
	Residual	8.60 (0.75), 7.24 to 10.21	N/A	8.58 (0.75), 7.22 to 10.18	N/A
	Intercept	26.79 (0.75), 20.77 to 34.57	N/A	22.52 (2.98), 17.37 to 29.20	N/A

^a^The number of observations in these models at level 1 (timepoint) is 417; at level 2 (participant) it is 155.

^b^DAU: days of app usage.

^c^ACEs: adverse childhood experiences.

^d^N/A: not applicable.

## Discussion

### Principal Findings

This study sought to assess whether the JoyPop app promotes changes in resilience-related outcomes in first-year undergraduate students over 4 weeks. In particular, this study focused on whether the JoyPop app would be helpful not just for those whose life experiences have paved the way for positive, adaptive development, but for youth whose histories of adversity put them at risk for lacking the foundational capacities that underlie resilience. Our hypotheses were partially supported. We found a dose-response relationship between days of app usage over the study and improvements in difficulties with emotion regulation and depression symptoms. Without asking for whom these changes are evident, it would remain unknown how this relationship is qualified by past adversity. Including ACEs score as a covariate in our models, we saw changes in difficulties in emotion regulation over time depending on one’s ACEs score. Specifically, those with higher ACEs scores had higher mean pre-app difficulties with emotion regulation, but faster rates of change such that the discrepancy with their lower-ACEs counterparts was reduced by the end of the study period. This is consistent with our expectation that those with more adversity have more to gain from an intervention. For depression symptoms, however, reductions with app usage did not differ depending on one’s ACEs score, indicating the app was seemingly effective for those with and without histories of adversity.

The main finding with respect to emotion regulation is in line with two claims from previous literature: first, that those with ACEs have poorer self-regulatory capacities [11], and second, that this can be restored through external supports [30,31]. Specifically, this finding is consistent with previous research that asserts a relationship between ACEs and poorer emotion regulation [13,14,75]. Moreover, the observation that both individuals with and without adversity responded to the intervention in terms of emotion regulation, albeit at different rates, supports the notion of resilience as a common process and the merit of bolstering basic adaptive capacities in all individuals. With respect to depressive symptoms, although the effect was not moderated by ACEs score, the finding of a dose-response relationship to app usage is also important considering university students face higher rates of depression [19], heightened distress compared to their pre-university levels [20], and the potential for suicidal ideation with high PHQ-9 scores [76]. Finding improvement in both emotion regulation and depression symptoms could also be viewed as consistent with prior research showing emotion regulation mediates the relationship between ACEs and negative mental health outcomes [13,14]. Specifically, considering the design of the app, reductions in depression may have been related to improved emotion regulation during the university transition. This possibility, however, was not formally tested in this study and could be a direction of future research.

This study contrasts with other intervention studies demonstrating improvements in resilience using the CD-RISC-10 [29] and the notion that resilience should be promoted when basic self-regulatory capacities (including executive functioning) are bolstered [22]. Instead, the JoyPop app did not impact self-reported resilience or executive functioning as hypothesized. At the same time, our findings could be construed as consistent with two alternate views suggested by research. First, if emotion regulation is one of the core components of resilience and plays a mediating role in this regard, then it should change first before more broad changes to general measures of resilience are seen. Consistent with this, Wright et al [22] highlighted that the effects of resilience interventions may take time to occur or may manifest indirectly, and as such, outcomes in multiple domains must be monitored over time. The same logic may be applied to depressive symptoms: emotion regulation is a potential mediating factor between childhood adversity and mental health difficulties like depression symptoms [4]. Second, an alternate view would hold that resilience and emotion regulation, although related, are not inextricably linked. The two constructs have indeed been operationalized as completely independent: for example, Poole et al [75] studied how the effect of emotion regulation on anxiety varies according to resilience scores, examining them separately in a moderating fashion as opposed to a mediating one. Although emotion regulation is indeed a component of resilience, the precise way in which resilience should change when emotion regulation changes has not been addressed by past research. A future direction could be to investigate at which point one’s overall trait-like resilience changes as a function of app-related changes in emotion regulation.

Another possibility is that many of the app features are more directly related to emotion regulation than to the domains that did not appear to change. For example, the Rate My Mood feature was meant to increase awareness of emotion, and the Breathing Exercises and Journal feature were meant to regulate emotion. Even SquareMoves, although meant to be more cognitively oriented, can be used as a means of emotion regulation in the form of providing a distraction. In fact, preliminary qualitative exploration of user experiences with the app found just this: many reasons for using certain features had to do with their impact on one’s emotions [77]. In addition to this, the Rate My Mood feature opened upon launching the app. Thus, this feature, which is directly tied to emotional capacities, may have been used more often than other features targeting other self-regulatory functions.

There was some consistency with previous literature in the relationship between ACEs and executive functioning. In the initial model with days of app usage, ACEs score, and the 2-way interaction between these variables, the only significant effect was for ACEs score, which suggests higher ACEs scores were associated with lower executive functioning overall. The relationship between ACEs and resilience, however, contrasted with previous research. In our sample, those with a relatively high number of ACEs scored no lower on the CD-RISC-10 resilience measure, on average, than those with fewer. One interpretation is that our sample could be considered resilient from the outset, considering they are doing well enough to attend university, which constitutes higher educational attainment than might be expected in those with difficult life circumstances. It could be that these individuals have succeeded academically because they have exercised resilience despite deficits in depressive symptoms or executive functioning that were associated with higher ACEs scores. For these students, adversity may not have been a barrier to developing resilience but instead functioned as the opposite, equipping them with the hardiness to deal with future stressors. This is consistent with stress-inoculation theory [23] and the challenge model of resilience [8]. Regardless of the reason, finding that the JoyPop app helps with emotion regulation and depression symptoms for a sample of students paves the way for future research to examine the impact of the app with more vulnerable populations.

### Strengths, Limitations, and Future Directions

This study had many methodological strengths. The use of MLM represents a sophisticated statistical approach that accounts for the nonindependence of longitudinal data. Inclusion of an individualized, time-varying covariate in the model (that is, days of app usage) allowed a direct dose-response link between app usage and outcomes. This contrasts with studies comparing post-intervention scores to pre-intervention scores with no consideration for individual differences in engagement with the intervention. Further, our intervention was unique in having few requirements for usage other than encouraging participants to try to use the app twice daily. This flexibility in allowing users to decide when, how often, and in what way to use the app more closely reflects app usage practices outside of a research context. Finally, an asset of this study is the inclusion of multiple facets of resilience, including its theoretical underlying processes and overall self-reported resilience.

In terms of limitations, the study lacked a control group of non–app users, which is a limitation of many app studies [41]. By using individual days of app usage over the study period as the metric of time, however, we are able to conclude that those using the app more evidenced more change than those using it a smaller proportion of days, with individuals who did not often use the app serving as a control group of sorts. Thus, while having a control group would represent the most convincing evidence in asserting that observed changes are due to app use, the relationship between days of app usage and the outcomes of interest helps refute the argument that changes were due to the passage of time. Still, participants who used the app more could have been more motivated to respond to successive questionnaires in a way that would demonstrate improvement.

As Lui et al [41] describe the lack of independent investigations and replications of results with respect to app-based interventions, further studies should attempt to replicate the findings outlined here to continue to build the evidence base for the JoyPop app. Moreover, exploring users’ experiences and satisfaction with the app will be important, as positive evaluations of the look, feel, and relevance of a smartphone app can influence ongoing use and engagement [78]. Additional research exploring the economic impact of app-based interventions is also required, as noted in two recent reviews [79,80]. For instance, it will be important to examine the cost-effectiveness and cost utility of integrating an intervention like the JoyPop app into usual care for youth struggling with their mental health. There also remains a need to evaluate long-term outcomes once app usage has ceased to determine whether ongoing usage is needed for maintained benefits or if improvements are sustained when the app is no longer in use. A meta-analysis of mental health–related smartphone apps could not assess this kind of sustainability because so few studies included long-term follow-up [38]. Having more timepoints in general would also allow nonlinear multilevel models to be tested, which could further describe the changes taking place. Finally, while data collection was staggered throughout the school year, another potential limitation is that students in our sample were at different stages of their transition to university. To better understand the impact of the JoyPop app on the transition to university, it would be ideal to recruit students within the first month of attendance.

A final limitation is the dearth of male participants in our sample, which prevented analysis of an interaction between ACEs score and sex in response to the app. Investigating the relationship between sex, adversity, and intervention response is important because there may be several potential sex differences, including rates of childhood adversity [81], neuroendocrine and brain responses to stress and trauma [82,83], susceptibility to certain psychological responses to adversity [84], and patterns of maturation of brain regions involved in stress regulation during adolescence [11]. The lack of male participants may have been a by-product of the relatively large number of psychology students enrolled, who tend to be female, but may also reflect a difference in interest in the app. Indeed, a meta-analysis of mental health interventions aimed at university students found only 24.7% of participants across all studies were male [85], which poses an important question about whether the needs of male students are being met with such interventions. A goal of future investigations will be to recruit a larger sample of male participants whose collective experiences span the range of possible ACEs scores.

In addition to the future research directions described thus far, the relative points of agreement and disagreement between our findings and previous research suggest future areas of study. First, addressing the relationship between emotion regulation and more distal outcomes, including depression and resilience, could be assessed using a longer-term study that may capture changes to the more trait-like CD-RISC-10. A mediation model could be explored to assess the precise way in which changes in emotion regulation might then relate to changes in resilience and depression later on. Further, it will be important to assess the effectiveness of the JoyPop app in a more vulnerable sample, for whom resilience may be a more pertinent construct. Lastly, measures mandated during the COVID-19 pandemic, including abrupt changes to education, mental health services, and social practices, have caused significant disruptions for youth [86]. Consequently, it has become increasingly important to optimize digital approaches to service delivery for youth, including smartphone apps, telemedicine, and virtual therapies [36]. As such, exploring whether the JoyPop app can aid in buffering against the negative effects of stress brought on by the current global pandemic is warranted.

### Conclusion

While the JoyPop app appears beneficial for emotion regulation and depressive symptoms, we found the effect on emotion regulation difficulties was qualified by the adversity participants had experienced during their childhood, such that those with ACEs improved the most and more quickly. The fact that those with ACEs were able to benefit in a similar fashion to their non–adversity-exposed counterparts speaks to the capacity for positive change within these individuals and the malleable nature of functions underlying resilience among youth. Importantly, not only did the features of the JoyPop app draw on ACEs research in promoting this positive change, but the smartphone medium of the intervention is an asset in terms of readily accessing help when it is needed. Our findings add to the growing literature on the importance of protecting and promoting self-regulatory capacities through well-timed interventions grounded in theory and research. The JoyPop app represents a positive step forward in catalyzing efforts to support resilience in youth with ACEs, particularly when it comes to regulating emotions during a time of transition.

## Appendix

Multimedia Appendix 1 Model-building steps and equations.

## Abbreviations

ACE: adverse childhood experience
CD-RISC-10: Connor-Davidson Resilience Scale–10
DERS-SF: Difficulties in Emotion Regulation – Short Form
MLM: multilevel modeling
PHQ-9: Patient Health Questionnaire–9
