# Supplementary material for: Adverse Childhood Experiences and Building Resilience With the JoyPop App: Evaluation Study
Source: JMIR Mhealth Uhealth. 2021 Jan 4;9(1):e25087. doi: 10.2196/25087 (PMC7813633; doi:10.2196/25087)
Supplement: Multimedia Appendix 1 [file mhealth_v9i1e25087_app1.docx]

**Model-Building Steps and Equations**

All MLMs were run using full information maximum likelihood estimation (FIML). Restricted maximum likelihood estimation (REML) provides less biased estimates of random effects (Bickel, 2007; Twisk, 2003) while FIML is considered superior for estimating fixed effects and must be used when hypothesizing about them (Twisk, 2003; Singer & Willett, 2003), although the differences between methods grows smaller as sample size increases. An independent residual covariance structure was specified for each model, which proved to be a good fit when comparing the Akaike and Bayesian information criterions to other residual covariance structures such as unstructured, autoregressive, and exchangeable.

First, the level 1 model was specified in its simplest form, the random intercept-only model, which is the most basic random effect that can be included in a multilevel model and accounts for the non-independence of repeated observations for each individual. Each individual’s outcome score *Y_ti_* equals their average outcome score, or intercept, β_0_*_i,_* and an error term *r_ti_* representing the deviation of the observed from predicted outcome. The intercept is equal to the grand mean of outcome scores across individuals (γ_00_) and the error term *u*_0i_, which is the deviation of the individual’s score from γ_00_.

Level 1 model: *Y_ti_ =* β_0_*_i_* + *r_ti_*

Where, at level 2: β_0_*_i_* = γ_00_ + *u*_0i_

The need for MLM was then assessed by examining the degree of variation in outcome scores across individuals, as suggested by Peugh (2010). The intraclass coefficient (ICC) and design effect were examined. For all outcomes, the ICC was much higher than zero and the design effect was greater than .20, suggesting MLM was appropriate. Time was then added as the only level 1 predictor. Specifically, time was operationalized as the number of days spent using the app as totaled at the baseline, midpoint, and the post-app sessions, where the maximum number of days the app could have been used would be 0, 14, and 28, respectively. This allows time to vary for each participant which is preferred to using the timepoint as the unit of time which would mask differences in actual app usage (Singer & Willett, 2003). The following equation, used as the first model for hypothesis testing, results:

*Y_ti_ =* β_0_*_i_* + β_1_*_i_*(*APP DAYS_ti_*) + *r_ti_*

Where, at level 2, in addition to the equation for β_0_*_i_* already expressed above:

β_1_*_i_* = γ_10_ + *u*_1i_

The intercept β_0_*_i_*_,_ the equation for which is expressed previously, becomes conditional on time such that it is now the outcome score at baseline. The added coefficient for app days, β_1_*_i_*, represents the rate of change over days of app usage, equal to the grand mean rate of change γ_10_ and a term reflecting individualized deviations in this slope *u*_1i_. This random slopes term is optional in MLM and a likelihood ratio test was used to assess whether model fit was improved for each outcome; this was the case for resilience and executive functioning only.

The level 2 models are created by adding level 2 predictors to the equations for β_0_*_i_* and β_1_*_i_*. Level 2 means the predictors exist at the individual level, as opposed to the time level, and are measured only at baseline. The first level 2 predictor added was ACE score, which formed a two-way interaction with days of app usage. Adding this covariate changes the interpretation of the parameters of the intercept and slope, as they represent the slope and intercept for those with no ACEs (ACE = 0). Likelihood ratio tests assessed fit improvement compared to the days of app usage-only models.

β_0_*_i_* = γ_00_ + γ_01_(*ACE_i_*) + *u*_0i_

β_1_*_i_* = γ_10_ + γ_11_(*ACE_i_*) *+ u*_1i_

At this point, additional covariates income and ethnicity were added to the model as main effects only, but they did not explain a significant amount of variation for any model.

The final model consisted of:

*Y_ti_* = γ_00_ + γ_01_(*ACE_i_*) + γ_10_ (*TIME_ti_*) + γ_11_(*ACE*)(*APP DAYS_ti_*) *+* *u*_0i_ *+ u*_1i_(*APP DAYS_ti_*) + *r_ti_*
